# Complete entry closure in isolated abdominal aortic dissection with a vascular plug: a case report

**DOI:** 10.1093/ehjcr/ytaf533

**Published:** 2025-10-14

**Authors:** Shin Hasegawa, Soh Hosoba, Akimitsu Tanaka, Takeki Ohashi

**Affiliations:** Department of Cardiology, Nagoya Tokushukai General Hospital, 2-52 Kozoji-cho Kita, Kasugai, Aichi 487-0016, Japan; Department of Cardiovascular Surgery, Nagoya Tokushukai General Hospital, 2-52 Kozoji-cho Kita, Kasugai, Aichi 487-0016, Japan; Department of Cardiology, Nagoya Tokushukai General Hospital, 2-52 Kozoji-cho Kita, Kasugai, Aichi 487-0016, Japan; Department of Cardiovascular Surgery, Nagoya Tokushukai General Hospital, 2-52 Kozoji-cho Kita, Kasugai, Aichi 487-0016, Japan

**Keywords:** Abdominal aortic aneurysm, Amplatzer Vascular Plug, Case report, Endovascular treatment, Isolated abdominal aortic dissection

## Abstract

**Background:**

Isolated abdominal aortic dissection (IAAD) is a rare vascular condition. Treatment options remain unclear due to the lack of established consensus on the most effective approach. While endovascular plugs have been successfully reported to seal the primary entry tear in thoracic aortic dissections, their utility in IAAD remains uncertain.

**Case summary:**

A 79-year-old woman presented with abdominal pain and was diagnosed with acute Stanford Type B aortic dissection, for which conservative blood pressure management was undertaken. A dissecting aneurysm in the infrarenal abdominal aorta and right common iliac artery (CIA) was identified. Follow-up computed tomography at 55 months revealed rapid aneurysmal expansion in the CIA. To prevent rupture, two intimal tears were sealed using an Amplatzer Vascular Plug II (AVP II; Abbott Laboratories, Chicago, IL, USA), resulting in complete elimination of contrast leakage into the false lumen and reduced aneurysm size in mid-term follow-up.

**Discussion:**

The use of the AVP in IAAD proved effective by promoting thrombosis within the false lumen and preventing its expansion. This offered a less invasive alternative, avoiding complications associated with endovascular aortic repair or open surgery, especially given the patient’s challenging anatomy. However, potential risks such as exacerbation of the entry tear or embolic events should be considered, warranting careful deployment with an endovascular plug. Amplatzer Vascular Plug-based entry closure provides an effective, less invasive strategy for IAAD management. Further research is needed to confirm its long-term safety and efficacy.

Learning points

- Using the Amplatzer Vascular Plug (AVP) for entry closure in expanding isolated abdominal aortic dissection (IAAD) may promote complete false lumen thrombosis, effectively preventing further expansion and potentially encouraging aneurysm reduction.
- The application of AVP in this case highlights a novel treatment strategy for IAAD that circumvents the risks associated with traditional endovascular aortic repair (EVAR) or open surgical intervention.
- This approach offers a promising alternative for cases with complex vascular anatomy.

## Introduction

In cases of expanding chronic aortic dissection, surgical or endovascular aortic repair (EVAR) may be unsuitable due to patient-specific anatomical or medical factors.^1^ For thoracic aortic dissections, endovascular devices that target and seal the primary entry tear have shown potential in closing the entry to the false lumen and promoting aortic remodelling.^2^ However, isolated abdominal aortic dissection (IAAD) is an uncommon entity with few previous reports.^3-5^ Notably, the Amplatzer Vascular Plug (AVP) (Abbott Laboratories, Chicago, IL, USA) use in IAAD has been limited, and its efficacy and safety remain unclear.

## Summary figure

**Figure:**
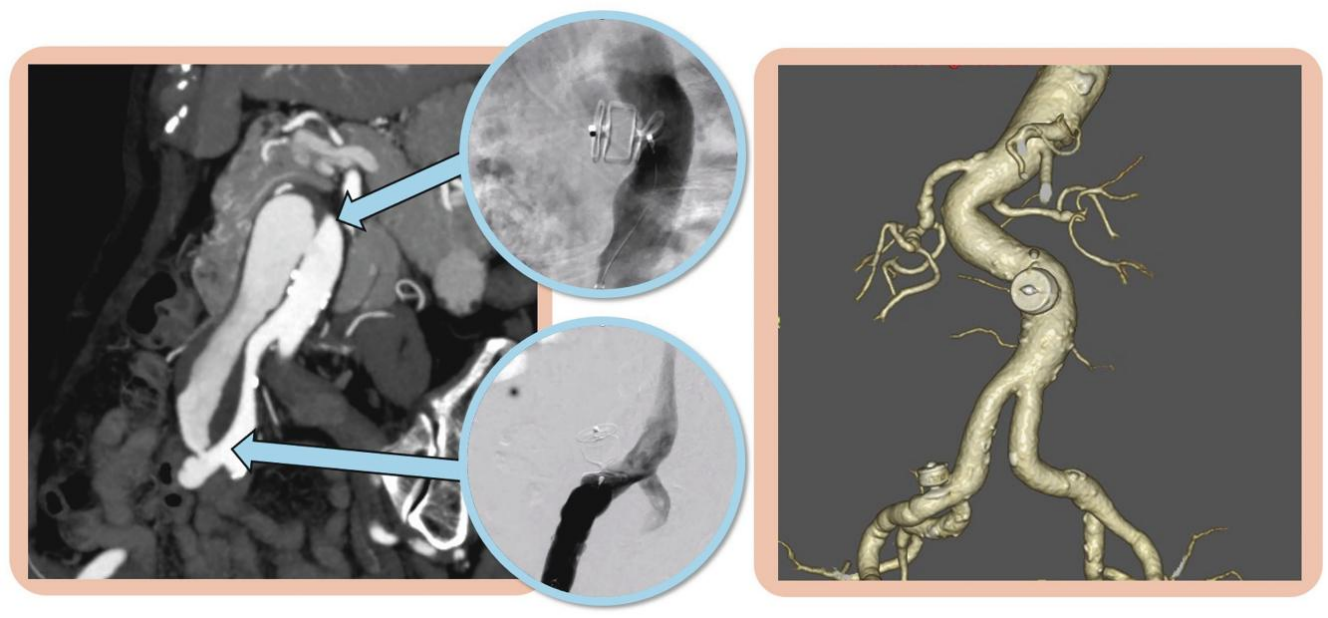


## Case presentation

**Figure 1:**
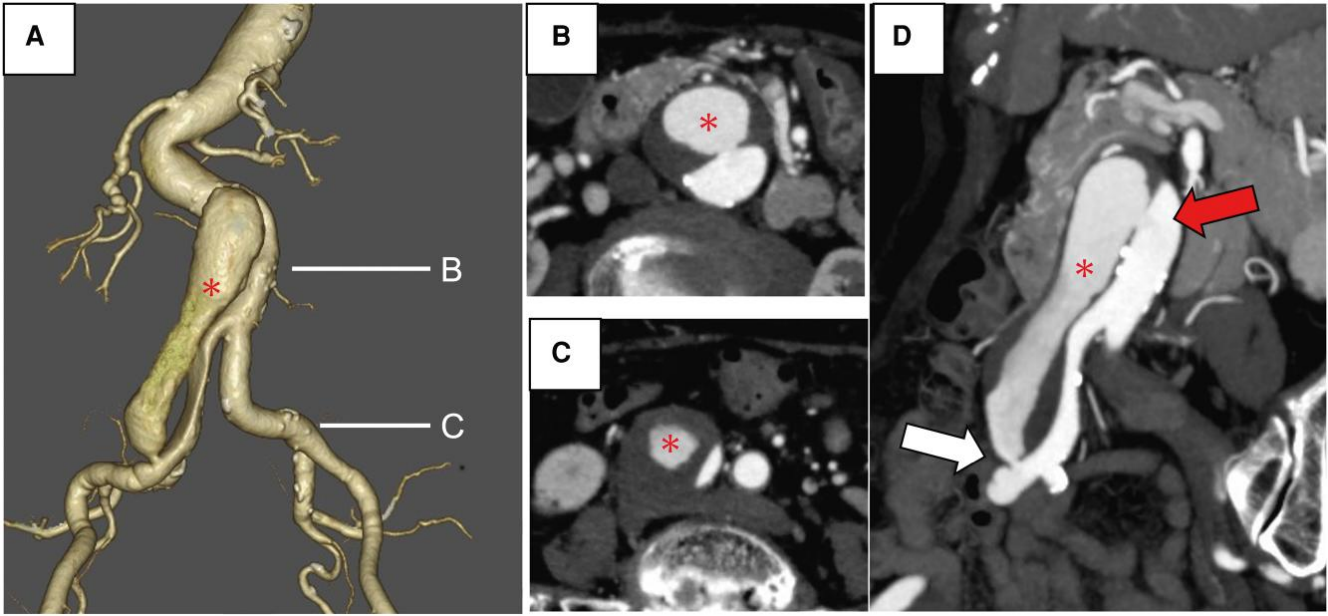
Contrast-enhanced computed tomography of the isolated abdominal aortic dissection. Volume rendering of the abdominal aorta shows a localized false lumen (asterisk) confined to the abdominal region (*A*). Notable findings include stenosis of the right common iliac artery and significant curvature of the infrarenal abdominal aorta. The axial images of the contrast-enhanced computed tomography demonstrate the site of the maximum diameter of the abdominal aortic aneurysm (*B*), the right common iliac artery aneurysm (*C*), and false lumen (asterisk). A 7 mm entry tear in the infrarenal abdominal aorta (superior arrow). Additionally, a 5 mm re-entry tear is noted in the right common iliac artery (inferior arrow) (*D*). CIA, common iliac artery; CT, computed tomography.

A 79-year-old woman with a history of hypertension presented to our institution with complaints of abdominal pain, leading to a diagnosis of acute Stanford Type B aortic dissection. She was not on any regular medications and no family history of cardiovascular disease. On physical examination, breath sounds were clear, heart sounds were normal with no murmurs, blood pressure was 145/73 mmHg, and heart rate was 86 b.p.m. Conservative management was initiated. Imaging revealed the presence of a dissecting aneurysm in the infrarenal abdominal aorta, as well as a right common iliac artery (CIA) aneurysm. The patient was under routine annual follow-up, and contrast-enhanced computed tomography (CCT) performed at 55 months demonstrated that the abdominal aortic aneurysm measured 3.8 cm, while the right CIA aneurysm exhibited a notable expansion from 2.9 to 3.5 cm over the preceding 6 months (*Video 1*). To prevent rupture, a multidisciplinary team, including vascular surgeons and cardiologists, evaluated various treatment options for the patient. Given her advanced age and frailty (clinical frailty scale 5), open surgical intervention was deemed to present a significant risk, and endovascular treatment was considered an appropriate approach. Contrast-enhanced CT imaging revealed significant angulation of the proximal landing zone in the abdominal aorta, suggesting a considerable risk of endoleak if EVAR is considered (*Figure 1A–C*). The primary entry tear was identified 5 cm below the renal artery in the abdominal aorta, with a size of 7 mm, while the re-entry tear was located 5 mm in the right CIA. The aortic dissection extended from 3 cm below the renal artery to the right CIA, with partial thrombosis observed in the false lumen. The lumbar artery and the inferior mesenteric artery branched from the true lumen. No additional entry tears were detected (*Figure 1D*).

**Figure 2:**
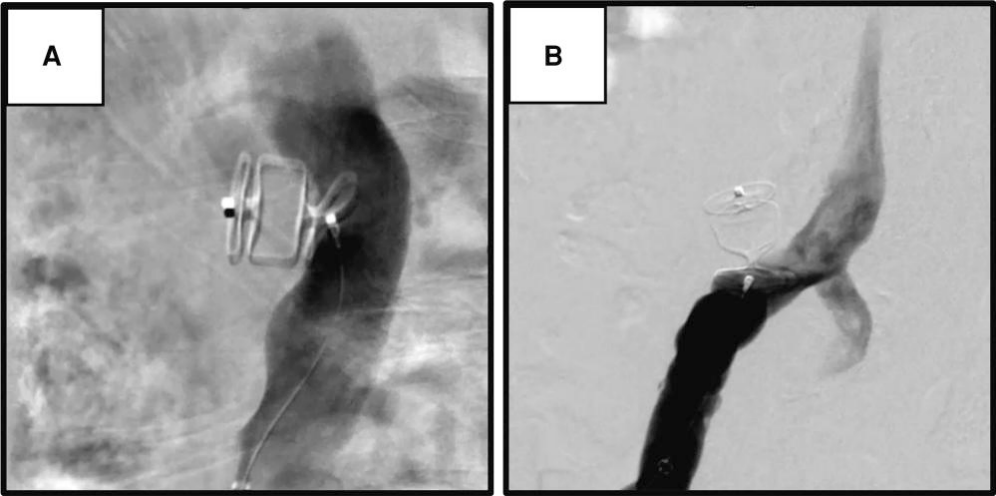
Angiography of endovascular treatment using Amplatzer Vascular Plug. The Amplatzer Vascular Plug was deployed in the entry site of the infrarenal abdominal aorta (*A*) and in the re-entry site of the right common iliac artery (*B*). In both regions, two discs were deployed in the false lumen, while one disc was positioned in the true lumen. AVP, Amplatzer Vascular Plug.

Considering the complexity of IAAD, the treatment strategy involved occluding the two tears using the AVP II to facilitate thrombus formation within the false lumen. If blood flow persists in the false lumen following this approach, a staged EVAR would be planned. The off-label use of endovascular plugs was approved by the institutional review board, with informed consent obtained from the patient. The procedure was performed under local anaesthesia. Access to both lumens was achieved via the femoral artery using a 6 Fr Destination Guiding Sheath (Terumo Corporation, Tokyo, Japan). An AVP II measuring 16 mm was deployed, with one disc positioned in the true lumen and two discs placed in the false lumen, effectively sealing the 7 mm intimal tear (*Figure 2A*). Subsequently, a 12 mm AVP II was deployed at the re-entry site, effectively sealing the 5 mm intimal tear (*Figure 2B*).

**Figure 3:**
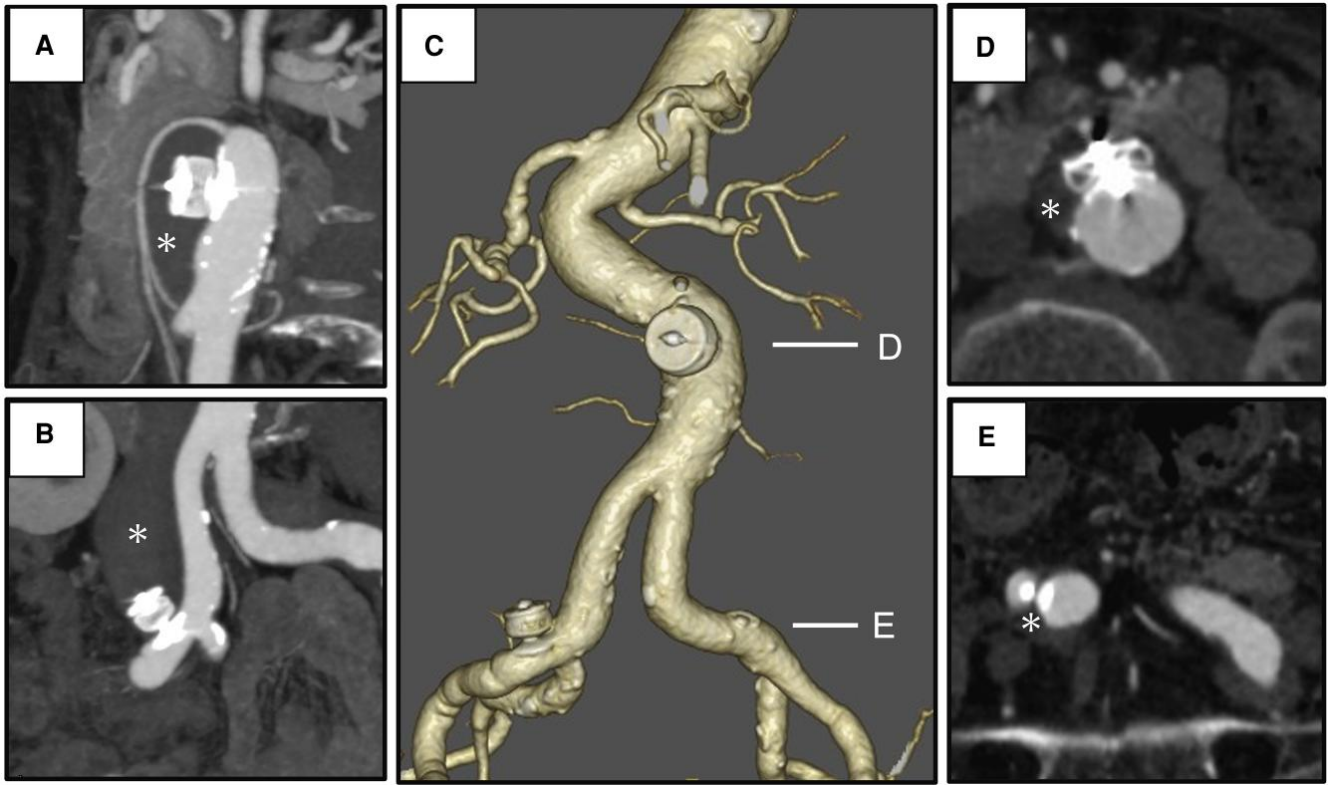
Follow-up contrast-enhanced computed tomography. A contrast-enhanced computed tomography performed 1 week after the procedure showed no blood flow into the false lumen (asterisk) of the abdominal aorta (*A*) and common iliac artery (*B*). Six months later, volume rendering confirmed no displacement of the two Amplatzer Vascular Plugs (*C*). The diameter of the infrarenal abdominal aorta reduced from 38 to 28 mm, while the right common iliac artery decreased from 34 to 22 mm. Additionally, the true lumen of the right common iliac artery expanded. Two years postoperatively, a computed tomography with contrast demonstrated significant regression of the false lumen (*D* and *E*). CIA, common iliac artery; CT, computed tomography.

The patient was discharged without complications on postoperative Day 1. A CT scan with contrast confirmed the absence of blood flow into the false lumen 1 week later (*Video 2*). At the 6-month follow-up, a CT imaging demonstrated successful repair of the intimal tear, with positive aortic remodelling, and the abdominal aortic aneurysm reduced to 28 mm and the right CIA aneurysm to 22 mm (*Figure 3A–C*). Two years post-treatment, follow-up CT showed no aneurysmal enlargement (*Figure 3D* and *E*). The true lumen diameter increased from 16 to 21 mm in the abdominal aorta and from 7 to 14 mm in the right CIA, while the false lumen diameter decreased from 22 to 7 mm in the abdominal aorta and from 28 to 12 mm in the CIA, indicating significant remodelling (*Table 1*).

**Table 1:** Timeline

Time	Event
Day 1	Patient presented with abdominal pain, diagnosed with acute Stanford Type B aortic dissection. Conservative management was performed.
Day 15	The patient was discharged without complications.
49 months	CT revealed a dissecting AAA (3.8 cm) and right CIAA (2.9 cm).
	CT showed a dissecting AAA (3.8 cm) and right CIAA (3.5 cm).
55 months	Elective procedure performed with AVP deployment to seal the intimal tears in the aorta and right common iliac artery.
1 week post-procedure	Follow-up CT confirmed the disappearance of blood flow to the false lumen.
2 years post-procedure	Follow-up CT demonstrated the absence of complications and a reduction in aneurysm size.

AAA, abdominal aortic aneurysm; CIAA, common iliac artery aneurysm; CT, computed tomography.

## Discussion

Isolated abdominal aortic dissection represents a rare vascular condition, constituting <2% of all aortic dissections.^1,3^ Treatment strategies for IAAD encompass optimal medical therapy (OMT), open surgical repair, and EVAR; nonetheless, there is no established consensus regarding the most effective approach.^2,4,5^ Patients with IAAD are commonly managed according to guidelines established for Stanford Type B aortic dissections, where invasive intervention is advised for aneurysms exceeding 5.5 cm in diameter or demonstrating an annual growth rate of more than 4 mm. For iliac artery aneurysms, a treatment threshold is typically set at 3.5 cm in diameter.^6^ In this case, although the IAAD itself measured <5 cm, the CIA aneurysm had reached a threshold of 3.5 cm, with rapid enlargement prompting timely intervention. Optimal medical therapy is considered both safe and feasible for low-risk IAAD patients, but mortality rates reported to reach as high as 41.6%, indicating that long-term outcomes may not always be favourable.^1^ For high-risk cases, open surgical repair is often recommended, with perioperative mortality rates reported to range between 20% and 30%. Conversely, EVAR has demonstrated high technical success rates and favourable clinical outcomes, supporting its role as a promising approach for the management of IAAD.^1^ Therefore, treatment selection should be individualized, with careful assessment of patient-specific risk factors and anatomical considerations to optimize therapeutic outcomes.

In thoracic aortic dissection, the safety and efficacy of closing the false lumen or entry tear using patent foramen ovale (PFO) or atrial septal defect (ASD) occluders, coils, endovascular plugs, stent grafts, and adhesives have been reported.^7,8^ Patients with a patent false lumen, particularly those with partial false lumen thrombosis, have been reported to have a higher mortality risk compared to those with a completely thrombosed false lumen.^9,10^ However, false lumen procedure (FLP) following thoracic endovascular aortic repair (TEVAR) has been associated with a complete thrombosis rate of up to 80%, potentially contributing to improved long-term outcomes.^8,9,10^ Reports have also highlighted the use of endovascular plugs as standalone treatments for FLP. The placement of an AVP at the site of the entry tear can maintain true lumen flow while promoting false lumen thrombosis and potentially mitigating aortic enlargement.^4,8,11^

The use of AVP in this case offers several advantages, including its occlusive effect on the intimal tear and its independence from branch vessel positioning, such as the renal arteries, and the size or morphology of access vessels. In contrast, in cases where the landing zone is angulated, EVAR may present an increased risk of endoleak or branch vessel occlusion.^12^ Additionally, in IAAD, the potential for stent graft-induced new entry tear (SINE) further underscores the complementary role of AVP in such cases. In the present report, entry closure for IAAD was successfully achieved using an appropriately sized AVPs that provided sufficient sealing, effectively preventing the progression of false lumen enlargement and facilitating aneurysm reduction. While extensively documented in thoracic aortic dissections, this report provides the first evidence of its efficacy within the abdominal aorta. However, the long-term outcomes of AVP treatment, particularly with respect to re-dissection and enlargement of the false lumen after entry closure, remain uncertain. Therefore, careful device selection and postoperative monitoring are critical, and further validation through larger, more robust cohorts is necessary to establish its broader applicability in clinical practice.

## Lead author biography


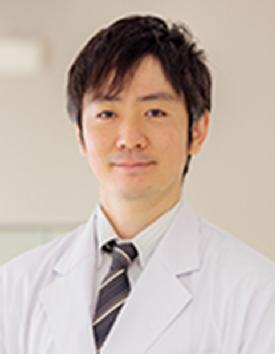


Shin Hasegawa graduated from Gifu University School of Medicine in 2015 and completed his initial training there until 2017. Since 2017, he has been working in the Department of Cardiology at Nagoya Tokushukai General Hospital. His interests within cardiovascular medicine include the treatment of structural heart disease, arrhythmias, and aortic diseases.

## Data Availability

The data that support the findings of this study are available from the corresponding author upon reasonable request. No restricted data were used in this study.
